# Neonatal microglia and proteinase inhibitors‐treated adult microglia improve traumatic brain injury in rats by resolving the neuroinflammation

**DOI:** 10.1002/btm2.10249

**Published:** 2021-08-27

**Authors:** Ping Zheng, Qingke Bai, Jiugeng Feng, Bing Zhao, Jian Duan, Lin Zhao, Ning Liu, Dabin Ren, Shufeng Zou, Wei Chen

**Affiliations:** ^1^ Department of Neurosurgery Shanghai East Hospital, School of Medicine, Tongji University Shanghai China; ^2^ Department of Neurology The People's Hospital of Shanghai Pudong New Area Shanghai China; ^3^ Department of Neurosurgery The First Affiliated Hospital of Nanchang University Nanchang China; ^4^ Department of Neurosurgery The People's Hospital of Shanghai Pudong New Area Shanghai China; ^5^ Department of Neurosurgery The First Affiliated Hospital of Nanjing Medical University Nanjing China

**Keywords:** adult microglia, neonatal microglia, protease inhibitor, traumatic brain injury

## Abstract

Microglia participate in the regulation of neuroinflammation caused by traumatic brain injury (TBI). This research aimed to explore the repair effects of intracranial injection of neonatal microglia or protease‐treated adult microglia on TBI in rat model. Lateral fluid percussion injury was used to establish rat brain injury model. E64 and serpinA3N were employed for the treatment of adult microglia. Cleaved caspase‐3 level was analyzed through immunoblotting assay. Enzyme‐linked immunosorbent assay was employed to analyze cytokine and chemokine levels. Astrocytosis and microgliosis were shown by immunofluorescence. The cognitive function of rats was analyzed by water maze. The injection of neonatal microglia inhibited cell apoptosis, reduced astrocytosis and microgliosis, decreased the level of chemokines and cytokines in cortex and ipsilateral hippocampus, and improved cognitive function of TBI rat model. The transplantation of peptidase inhibitors‐treated adult microglia also inhibited cell apoptosis, reduced astrocytosis and microgliosis, and improved cognitive function of rats with TBI. The transplantation of either neonatal microglia or peptidase inhibitors‐treated adult microglia significantly inhibited the pathogenesis of TBI in rat model, while untreated adult microglia showed no significant effect.

## 1 INTRODUCTION

Traumatic brain injury (TBI) is defined as brain tissue damage caused by external forces, which is a worldwide public health issue.^1^ TBI results in primary and secondary damage to the tissue of brain.^2^ The secondary damage of TBI has strong correlation with post‐TBI molecular mechanisms and results in neuroinflammation, oxidative damage, cytokine damage, excitotoxicity, and cell death.^3^ Researches have proven that several diseases are related to long‐term TBI pathophysiology, including Parkinson disease, Alzheimer disease, epilepsy, and post‐traumatic stress disorder.^4^

Under pathological conditions such as trauma, enhanced inflammation aggravates tissue damage.^5^ The inflammatory response of TBI is once thought to be caused by peripheral immune mediators passing through the damaged blood–brain barrier. Recent study illustrates that neuroinflammation occurs through a complex interaction between different kinds of cells and characterized by peripheral leukocyte recruitment and migration, resident cell activation, and inflammatory mediator release.^6^ Microglia play critical roles in neuroinflammation.^7^ Resident microglia initiate inflammatory cascade upon injury and microgliosis‐related proteins are typical TBI biomarkers.^8^ The responses of microglia in TBI include chemokines, inflammatory cytokines, and growth factors secretion, cellular debris and dying cell phagocytosis, cellular response modulation, and the formation of barrier around lesional area.^9^

Recently, research has proved that, in adult mice with spinal cord injury, both neonatal microglia and peptidase inhibitors‐treated adult microglia transplantation significantly promoted spinal cord repair.^10^ This result suggests that this therapeutic strategy can be employed to accelerate scar‐free healing. Since microglia are also involved in the pathogenesis of TBI, this strategy may also be efficiency in the alleviation of TBI‐caused damage. In this research, we aimed to explore the repair effects of intracranial injection of neonatal microglia and protease‐treated adult microglia on TBI in rat model.

## 2 METHODS

### 2.1 Establishment of rat TBI model

Lateral fluid percussion injury was used to establish rat brain injury model. Male rats were used in all experiments. After being fixed on a stereoscopic experimental table, the rats were anesthetized through inhaling isoflurane. In the middle of the head, the skin of rat was cut to expose the right parietal bone. Using a craniotomy, drill to make a hole 3 mm from the sagittal suture and 3.5 mm behind the coronal suture. Placed a small cap on the dura mater through the hole and fixed hydraulic tube to give 3 atmosphere (atm) overpressure (the standard for severe head injury). Rats in the sham operation group were only opened the bone window. Animal studies were approved by the Ethical Committee of the First Affiliated Hospital of Nanchang University.

### 2.2 Microglia isolation and transplantation

C57BL/6 rats were anesthetized through xylazine (10 mg/kg) and ketamine (100 mg/kg) and perfused with cold phosphate‐buffered saline (PBS) for the isolation of microglia. Cerebral cortices without dura were dissected from sex‐segregated neonates (postnatal day 1, P1) or adult rat (2 months) brain. Tissues were digested by Neural Tissue Dissociation Kit (P) (Miltenyi Biotec, Auburn, CA, USA). Resuspended dissociated cells in PBS with 0.5% bovine serum albumin (Invitrogen, Carlsbad, CA, USA) and filtered these cells through cell strainer (70 μm) (BD Biosciences, San Jose, CA, USA). In cell suspensions, myelin was depleted through myelin removal beads II and MACS system (Miltenyi Biotec) and microglial cells were isolated by CD11b MicroBeads and MACS system (Miltenyi Biotec).

Membrane‐permeable irreversible cysteine peptidase inhibitor E64 and serine protease inhibitor serpinA3N were used in this research. Adult microglial cells (1 × 10^6^ cell/ml) were treated with 100 ng/ml serpianA3N and 10 μΜ E64 for 30 min and then resuspended as previously described.^10^ Before transplantation, microglia cells were resuspended to around 2 × 10^5^ cell/ml. Vehicle, P1 microglia, adult microglia, or adult microglia (5 μL) treated by proteinase inhibitors were slowly injected through nanolitre injector (Nanoliter 2000, WPI, Stevenage, UK) 1 h after the induction of TBI. During transplantation, 30 μΜ E64 and 500 ng/mL serpinA3N were added again to proteinase inhibitors‐treated adult microglia.

### 2.3 Immunoblotting assay

Injured cortex and ipsilateral hippocampus were harvested for immunoblotting assay 5 days post injury. Tissues were lysed through RIPA buffer (Beyotime, Nantong, China) and centrifuged to get protein sample. Western blot was performed by the standard method. Membranes were separately incubated with anti‐β‐Actin (60008‐1‐Ig) (ProteinTech, Chicago, IL, USA) and anti‐Cleaved Caspase‐3 (9661) (CST, Danvers, MA, USA) at 4°C overnight and then incubated with peroxidase‐linked secondary antibodies at room temperature for 1 h. ECL Substrate Kit (Abcam, Cambridge, UK) was used to visualize protein blots and gray value was calculated by ImageJ.

### 2.4 Enzyme‐linked immunosorbent assay (ELISA)

Injured cortex and ipsilateral hippocampus were harvested for ELISA. The levels of interleukin 1β (IL‐1β), tumor necrosis factor α (TNF‐α), macrophage colony‐stimulating factor (Csf1), and monocyte chemoattractant protein (MCP)‐1 were simultaneously analyzed using kits obtained from Millipore (Millipore Milliplex, cat no. RCYTOMAG‐80K‐23; Billerica, MA, USA) on a MAGPIX instrument (Luminex Technologies; Luminex, Austin, TX, USA) according to the manufacturer's instructions.

### 2.5 Immunofluorescence

Rats were sacrificed through anesthesia 5 days post‐injury and perfused transcardially with PBS and 1% paraformaldehyde (PFA) successively. Fixed the brain tissues in 1% PFA for 12 h and 30% sucrose for 3 days at 4°C, then embedded in optimal cutting temperature compound (OCT), stored at −80°C. Sections were cut at 10‐μm thickness and prepared for immunofluorescence. The primary antibodies used were: anti‐GFAP (Z0334) (DAKO, Hamburg, Germany) and anti‐Iba1 (10904‐1‐AP) (ProteinTech). Secondary antibodies included: Alexa Fluor 488‐conjugated donkey anti rabbit (A‐21206) (Invitrogen, Carlsbad, CA, USA) and Alexa Fluor 555‐conjugated donkey anti rabbit (A‐31572) (Invitrogen). Slices were analyzed via confocal microscopy. Cell numbers of microglia and astrocytes in a HPF (high power field) of 200× view on the focus of lesion were counted artificially.

### 2.6 Morris water maze test

Morris water maze test was employed to evaluate the spatial learning and memory functions of rats. Morris water maze test was performed as described previously.^11^ Rats were trained in a water maze with a diameter of 210 cm for 5 days. During training, each rat underwent three trials. During each test, the rats were placed in different positions of the maze and allowed to find the platform for 90 seconds. After the test, the rats that failed to find the platform were placed on the platform. On the sixth day of the test, two trials were performed. During the first trial, each rat was placed in the quadrant opposite the platform and allowed 90 s to search the platform. Record the path of each rat in the water maze and the time it takes to locate the platform. The second test is a detection test after removing the platform. During the exploration test, the rats were placed in a water maze and allowed to explore for 30 s. The number of times each rat crossed the platform was recorded.

### 2.7 Statistical analysis

All statistical analyses were performed by SPSS 16.0 statistical software (SPSS Inc., Chicago, IL, USA). Data were shown as mean ± SEM. Statistical differences among groups were evaluated by one‐way ANOVA analysis with a Tukey's post hoc test; *p < 0.05, **p < 0.01, ***p < 0.001, ****p < 0.0001, ns means no significance.

## 3 RESULTS

### 3.1 P1 microglia infusion inhibited cell apoptosis in injured cortex and ipsilateral hippocampus

**FIGURE 1:**
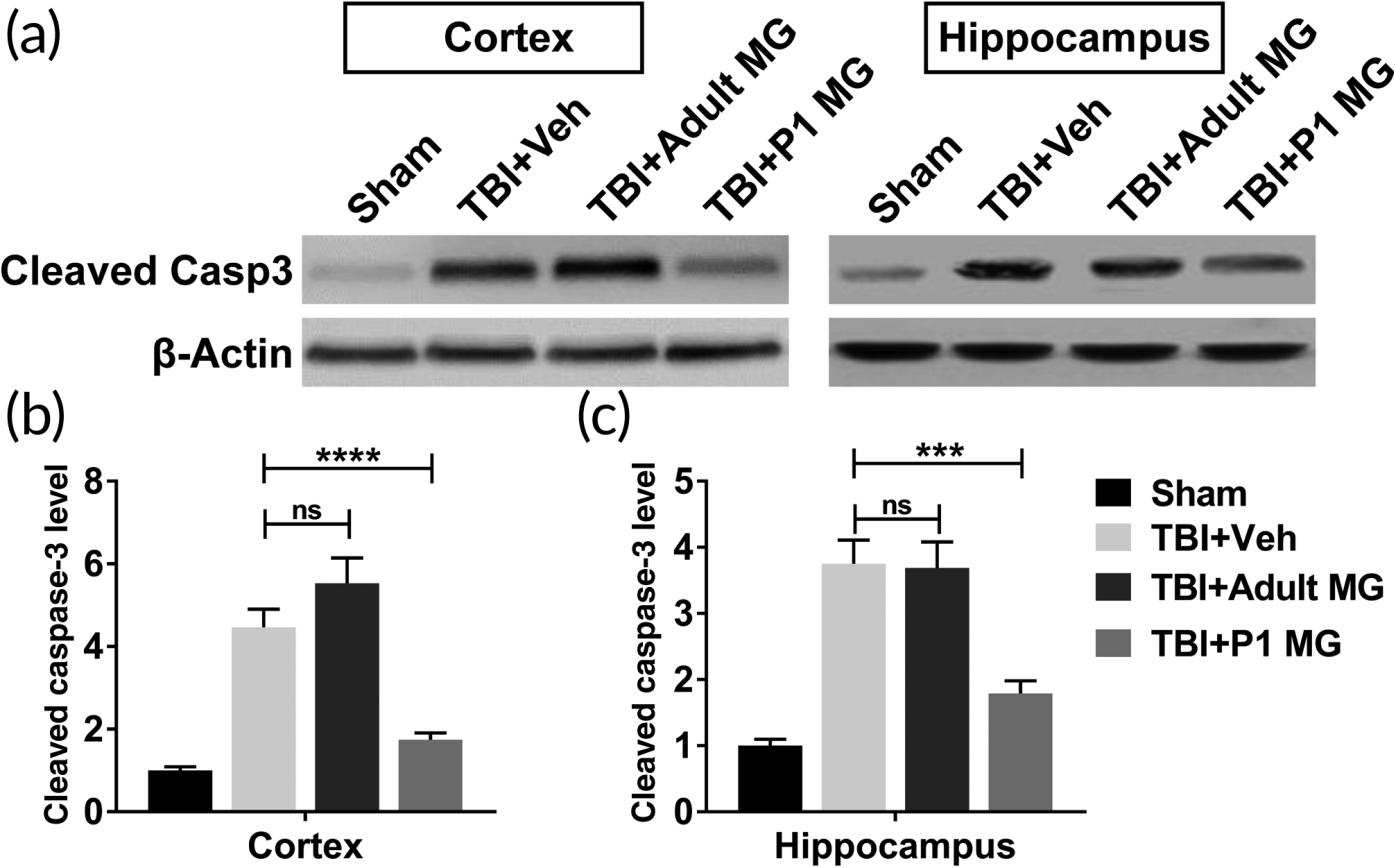
P1 but not adult microglia infusion inhibited cell apoptosis in injured cortex and ipsilateral hippocampus. Adult rats were conducted lateral fluid percussion injury or sham operation, subsequently with intracranial injection of vehicle (Veh), 1 × 10^6^ adult microglia, or 1 × 10^6^ P1 microglia. Injured cortex and ipsilateral hippocampus were harvested for immunoblotting assay 5 days post injury. The protein level of cleaved caspase‐3 of injured cortex (a, b) and ipsilateral hippocampus (a, c) was determined. *n* = 8. Data were presented as mean ± SEM. ***p < 0.001, ****p < 0.0001, ns, no significance

To investigate whether P1 microglia or adult microglia injection could inhibit cell apoptosis in the brain of rats with TBI, the protein levels of cleaved caspase‐3 were evaluated by immunoblotting assay. As shown in Figure 1a–c, when compared with rats treated by sham operation, rats with TBI which were injected with vehicle or adult microglia had significantly higher cleaved caspase‐3 protein levels. Meanwhile, cleaved caspase‐3 protein levels between TBI + vehicle group and TBI + adult microglia group showed no obvious difference (Figure 1a–c). However, elevated cleaved caspase‐3 protein levels in rats with TBI were dramatically declined by the injection of P1 microglia (Figure 1a–c). These results indicated that the adult microglia had no influence on TBI‐induced cell apoptosis, while P1 microglia alleviated cell apoptosis in brain.

### 3.2 P1 microglia infusion inhibited astrocytosis and microgliosis in injured cortex and ipsilateral hippocampus

**FIGURE 2:**
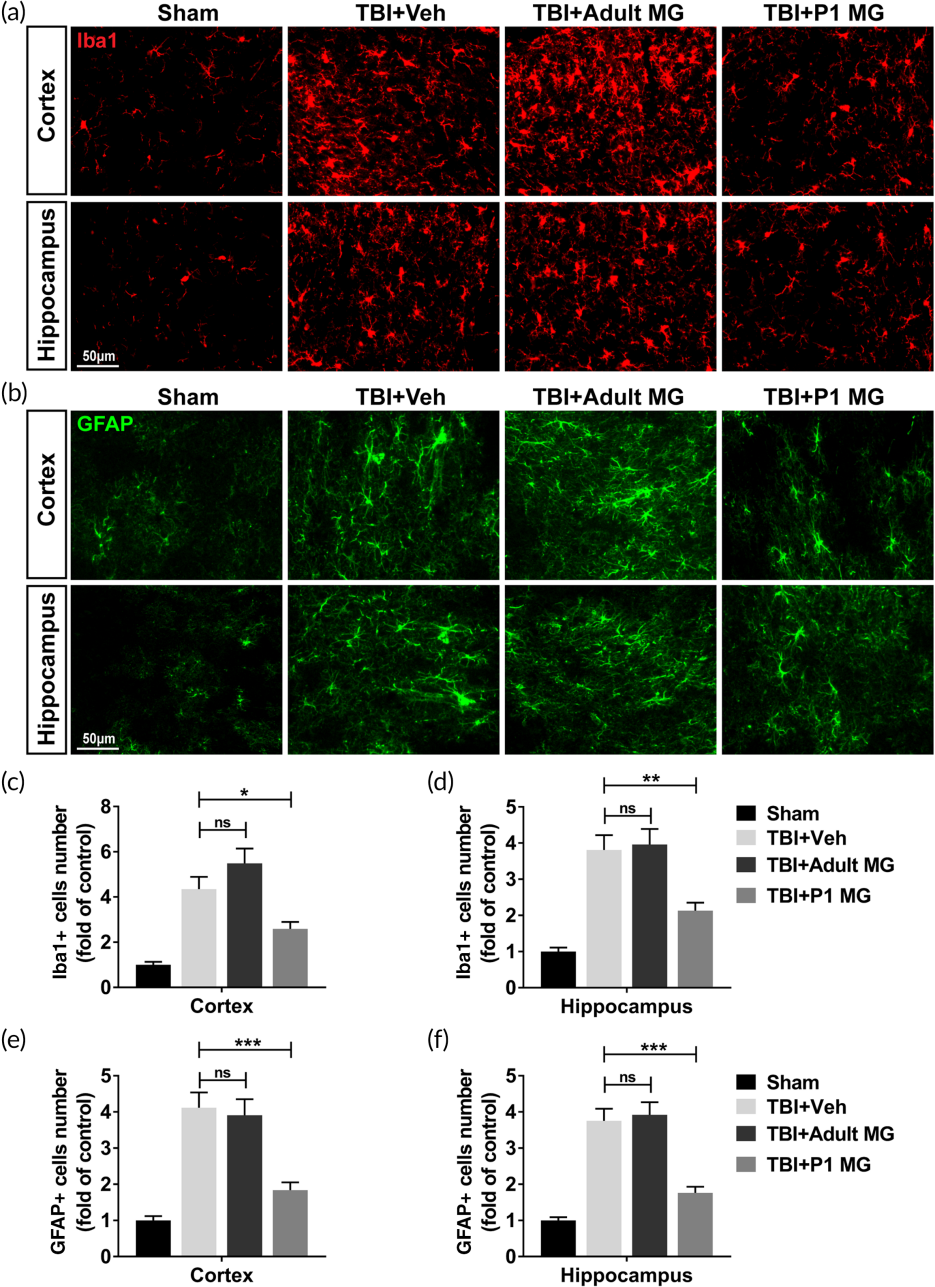
P1 but not adult microglia infusion reduced astrocytosis and microgliosis in injured cortex and ipsilateral hippocampus. Adult rats were conducted lateral fluid percussion injury or sham operation, subsequently with intracranial injection of vehicle (Veh), 1 × 10^6^ adult microglia, or 1 × 10^6^ P1 microglia. Injured cortex and ipsilateral hippocampus were harvested for immunofluorescent staining of Iba1 and GFAP 5 days post injury. (a, c, d) Microglia (Iba1+) in injured cortex (a, c) and ipsilateral hippocampus (a, d). (b, e, f) Astrocytes (GFAP+) in injured cortex (b, e) and ipsilateral hippocampus (b, f). *n* = 8. Data were presented as mean ± SEM. *p < 0.05, **p < 0.01, ***p < 0.001, ns, no significance

Astrocytes and microglia are crucial for the initiation of inflammatory response in brain. Proteins related to astrocytosis and microgliosis are employed as TBI biomarkers. In this research, microglia marker Iba1 and astrocytes marker GFAP were analyzed through immunofluorescence to show the influence of microglia injection on astrocytes and microgliosis activation. In TBI rat model, elevated Iba1+ microglia number in injured cortex and ipsilateral hippocampus was not influenced by the injection of adult microglia, but significantly decreased by the injection of P1 microglia (Figure 2a, c, d). Simultaneously, P1 microglia also declined GFAP+ astrocytes number in rat with TBI, while adult microglia showed no effect (Figure 2b, e, f). Thus, P1 microglia treatment inhibited astrocytosis and microgliosis in TBI rat brain.

### 3.3 P1 microglia infusion decreased cytokine and chemokine levels in injured cortex and ipsilateral hippocampus

**FIGURE 3:**
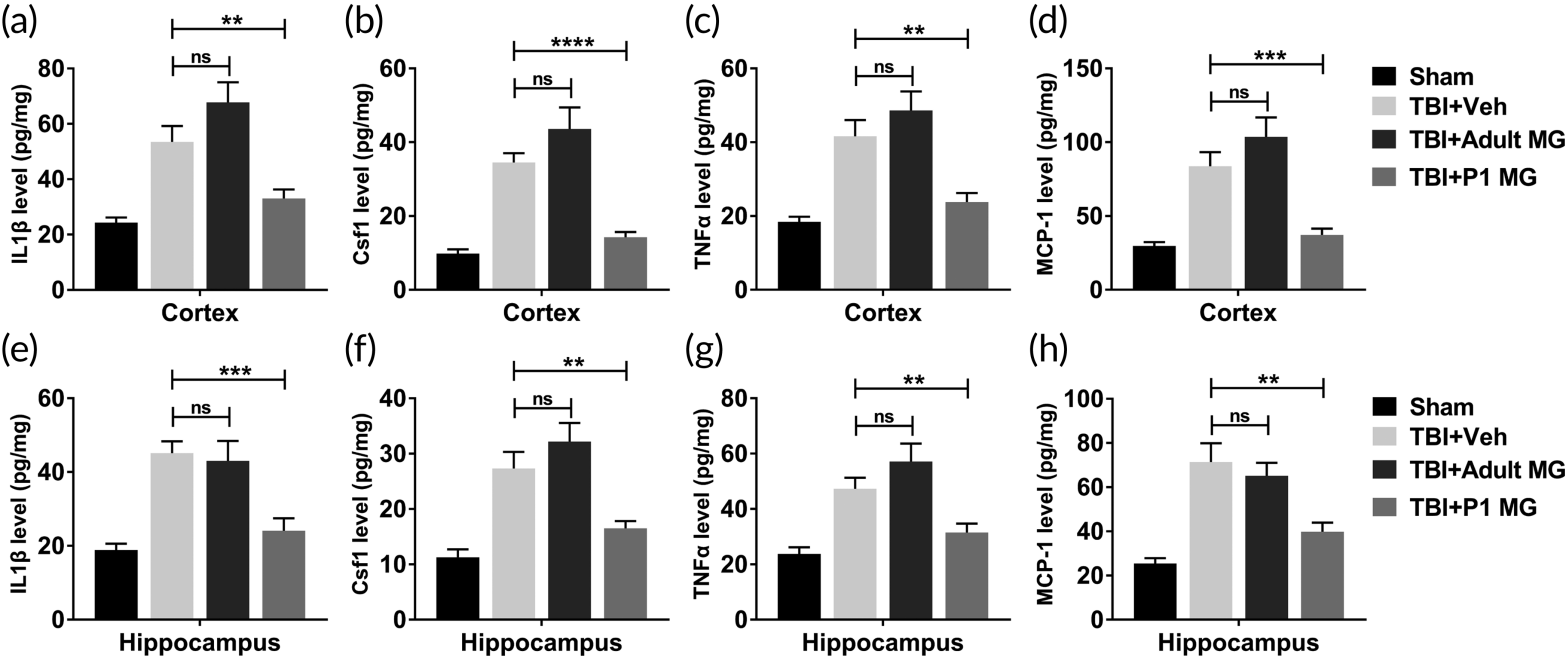
P1 but not adult microglia infusion decreased the level of cytokines and chemokines in injured cortex and ipsilateral hippocampus. Adult rats were conducted lateral fluid percussion injury or sham operation, subsequently with intracranial injection of vehicle (Veh), 1 × 10^6^ adult microglia, or 1 × 10^6^ P1 microglia. Injured cortex and ipsilateral hippocampus were harvested for ELISA 5 days post injury. (a–d) The protein level of IL‐1β (a), Csf1 (b), TNF‐α (c), and MCP‐1 (d) in injured cortex. (e–h) The protein level of IL‐1β (e), Csf1 (f), TNF‐α (g), and MCP‐1 (h) in ipsilateral hippocampus. *n* = 8. Data were presented as mean ± SEM. **p < 0.01, ***p < 0.001, ****p < 0.0001, ns, no significance

To further assess the inflammatory response in TBI rat model, the homogenates of injured cortex and ipsilateral hippocampus from rats in different group were analyzed for different cytokine/chemokines. In injured cortex, IL‐1β, Csf1, TNF‐α, and MCP‐1 levels were all elevated by the induction of TBI (Figure 3a–d). When compared with TBI rats treated with vehicle or adult microglia, those injected with P1 microglia had significantly lower IL‐1β, Csf1, TNF‐α, and MCP‐1 levels (Figure 3a–d). Meanwhile, the same phenomenon was also observed in ipsilateral hippocampus of rats (Figure 3e–h). The altered level of cytokines and chemokines indicated that P1 microglia treatment inhibited the inflammatory response in TBI rat model.

### 3.4 P1 microglia infusion improved the cognitive function after the induction of TBI

**FIGURE 4:**
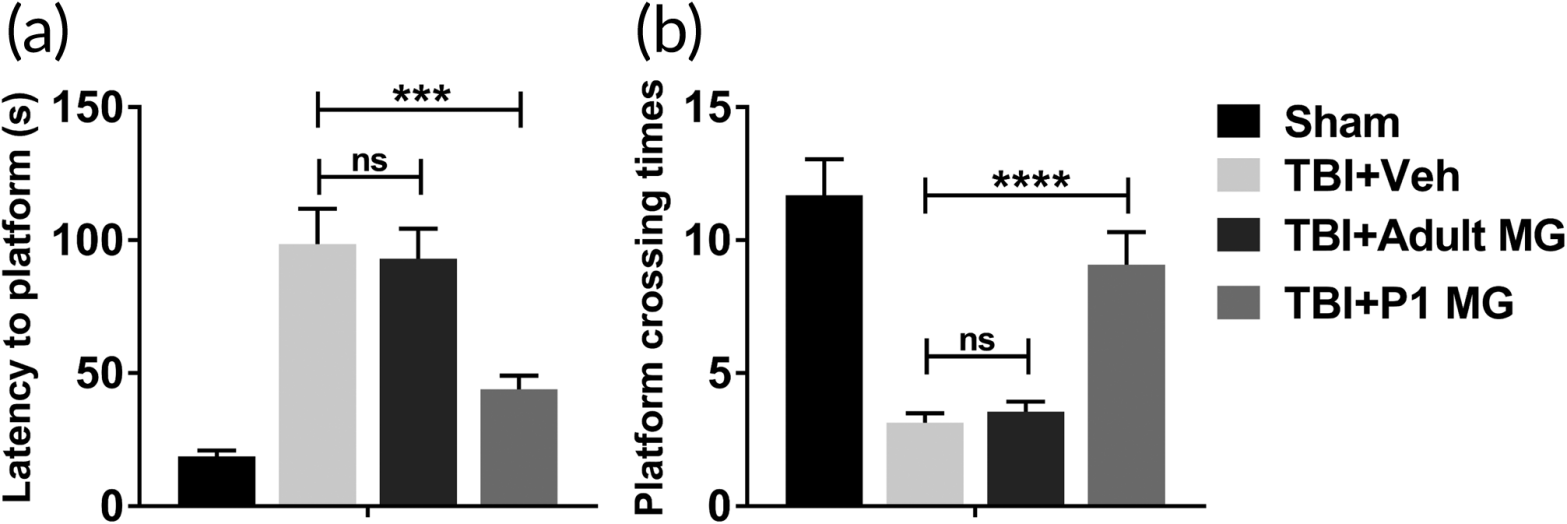
P1 but not adult microglia infusion improved cognitive function after TBI. Adult rats were conducted lateral fluid percussion injury or sham operation, subsequently with intracranial injection of vehicle (Veh), 1 × 10^6^ adult microglia, or 1 × 10^6^ P1 microglia. Water maze was performed to determine the cognitive function. Rats were trained for 5 days and examined at sixth day. (a) Escape latency to the platform was quantified. (b) The platform was removed and the times of platform location crossing in 5 min were recorded. *n* = 20. Data were presented as mean ± SEM. ***p < 0.001, ****p < 0.0001, ns, no significance

In Figure 4a, b, adult microglia injection had no significant influence on increased escape latency and decreased platform crossing times of rats with TBI. However, both altered escape latency and platform crossing times induced by the induction of TBI were rescued by the injection of P1 microglia (Figure 4a, b). The performance of rats in water maze demonstrated that P1 microglia improved cognitive function of rats after the induction of TBI.

### 3.5 Protease inhibitors‐treated adult microglia infusion inhibited cell apoptosis in injured cortex and ipsilateral hippocampus

**FIGURE 5:**
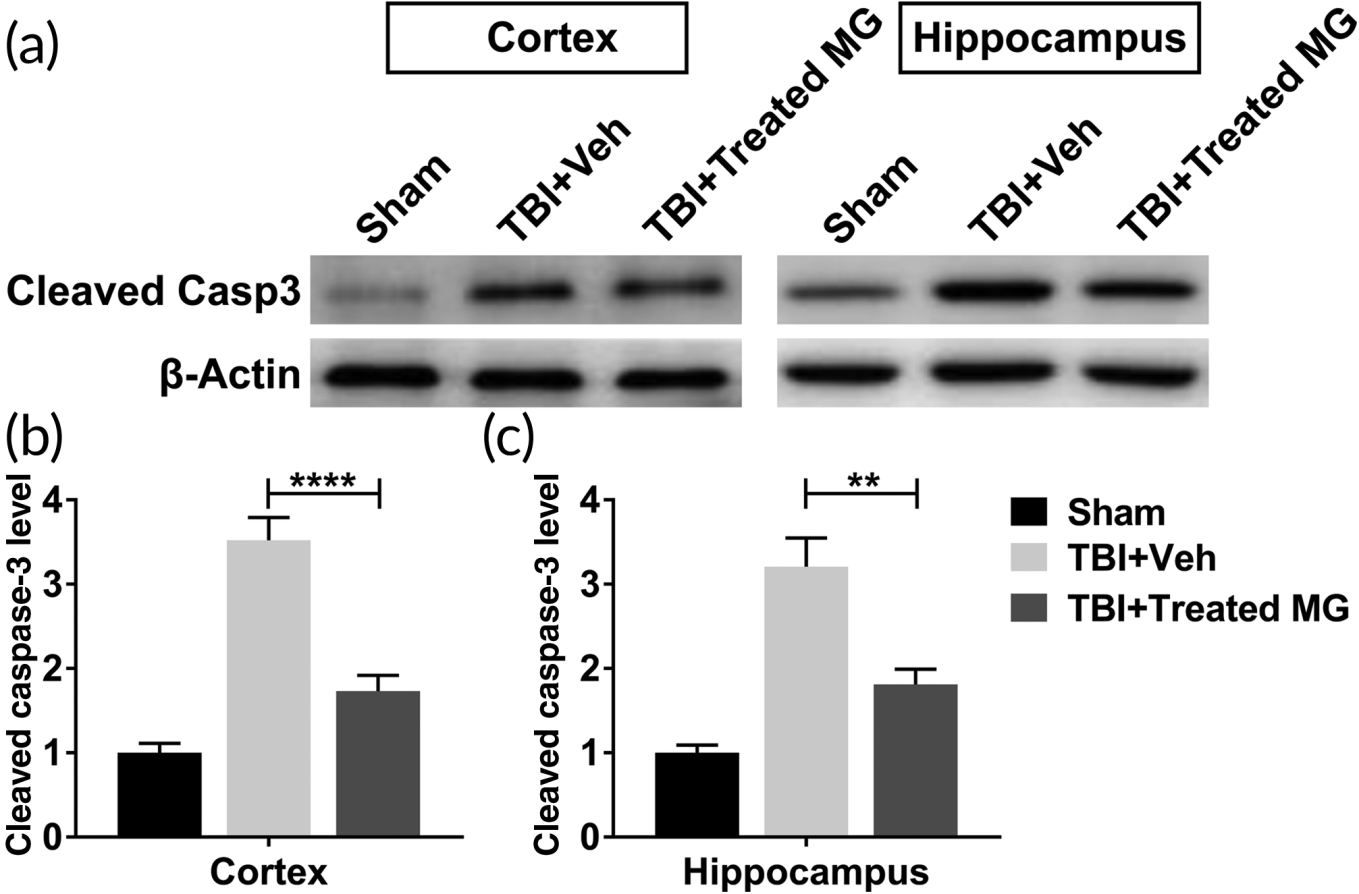
Protease inhibitors‐treated adult microglia infusion inhibited cell apoptosis in injured cortex and ipsilateral hippocampus. Adult rats were conducted lateral fluid percussion injury or sham operation, subsequently with intracranial injection of vehicle (Veh) or 1 × 10^6^ E64‐ and serpinA3N‐treated adult microglia. Injured cortex and ipsilateral hippocampus were harvested for immunoblotting assay 5 days post injury. The protein level of cleaved caspase‐3 of injured cortex (a, b) and ipsilateral hippocampus (a, c) was determined. *n* = 9. Data were presented as mean ± SEM. **p < 0.01, ****p < 0.0001

In this research, we had proved that the injection of adult microglia had no effects in alleviating the pathogenesis of TBI in rat model. Whether protease inhibitors‐treated adult microglia could inhibit TBI was explored. As shown in Figure 5a–c, elevated protein level of cleaved caspase‐3 caused by the induction of TBI was significantly decreased by the injection of protease inhibitors‐treated adult microglia. Thus, the administration of adult microglia treated by E64 and serpinA3N alleviated cell apoptosis in rats with TBI.

### 3.6 Inhibitors‐treated adult microglia infusion inhibited astrocytosis and microgliosis in injured cortex and ipsilateral hippocampus

**FIGURE 6:**
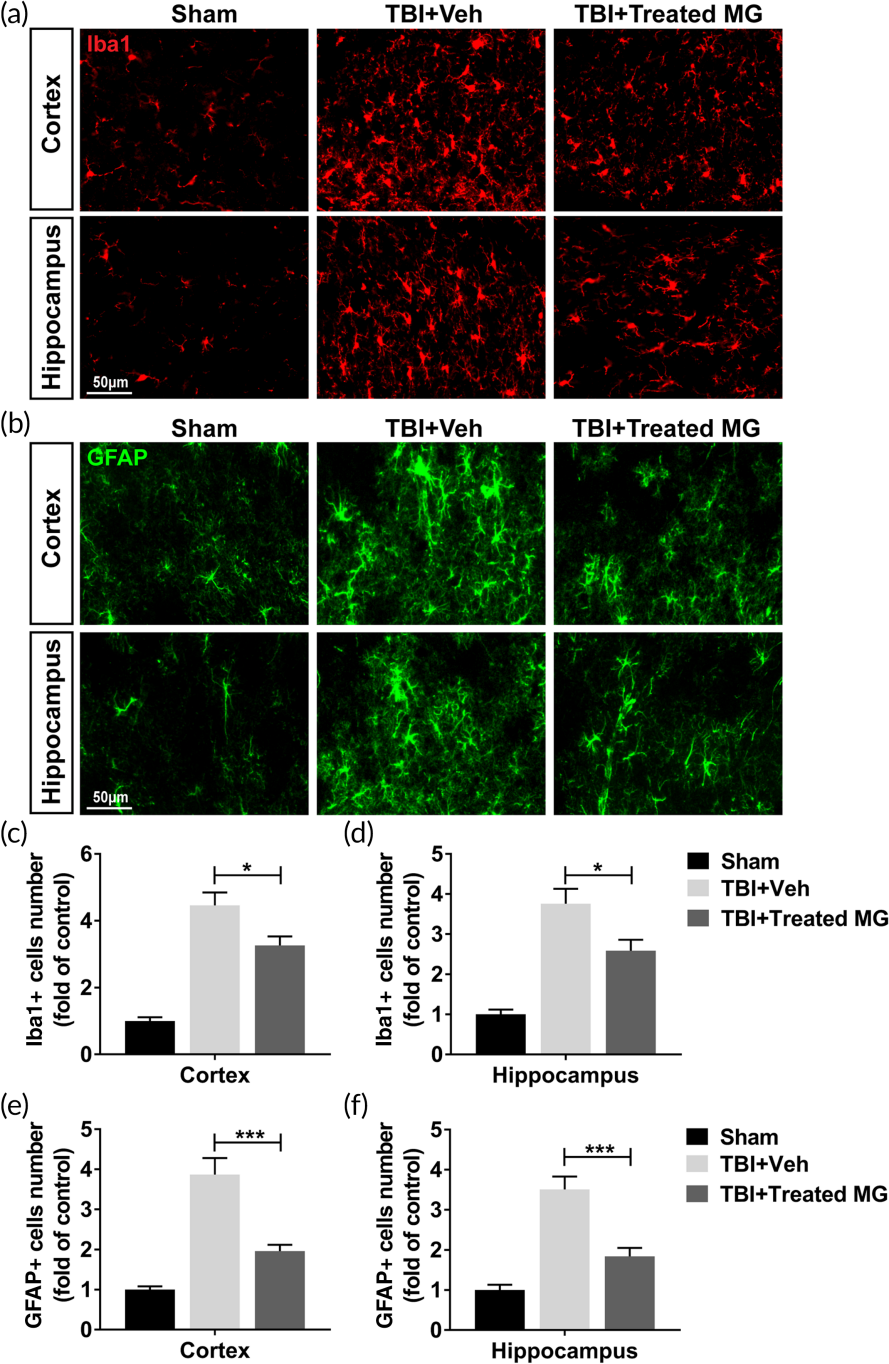
Protease inhibitors‐treated adult microglia infusion reduced astrocytosis and microgliosis in injured cortex and ipsilateral hippocampus. Adult rats were conducted lateral fluid percussion injury or sham operation, subsequently with intracranial injection of vehicle (Veh) or 1 × 10^6^ E64‐ and serpinA3N‐treated adult microglia. Injured cortex and ipsilateral hippocampus were harvested for immunofluorescent staining of Iba1 and GFAP 5 days post injury. (a, c, d) Microglia (Iba1+) in injured cortex (a, c) and ipsilateral hippocampus (a, d). (b, e, f) Astrocytes (GFAP+) in injured cortex (b, e) and ipsilateral hippocampus (b, f). *n* = 9. Data were presented as mean ± SEM. *p < 0.05, ***p < 0.001

Furthermore, we also investigated the effect of inhibitors‐treated adult microglia on astrocytosis and microgliosis in TBI rat model. In adult rat brain, Iba1+ microglia number in TBI rat treated with inhibitors‐treated adult microglia was significantly lower than those treated with vehicle (Figure 6a, c, d). Simultaneously, the injection of inhibitors‐treated adult microglia also significantly declined the number of GFAP+ astrocytes (Figure 6b, e, f). These results proved that the treatment of E64 and serpinA3N improved the effect of adult microglia on inhibiting astrocytosis and microgliosis in TBI rat model.

### 3.7 Protease inhibitors‐treated adult microglia infusion improved the cognitive function after the induction of TBI

**FIGURE 7:**
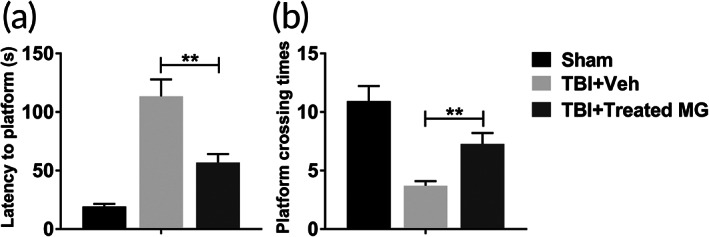
Protease inhibitors‐treated adult microglia infusion improved cognitive function after TBI. Adult rats were conducted lateral fluid percussion injury or sham operation, subsequently with intracranial injection of vehicle (Veh) or 1 × 10^6^ E64‐ and serpinA3N‐treated adult microglia. Water maze was performed to determine the cognitive function. Rats were trained for 5 days and examined at sixth day. (a) Escape latency to the platform was quantified. (b) The platform was removed and the times of platform location crossing in 5 min were recorded. *n* = 20. Data were presented as mean ± SEM. **p < 0.01

The effect of protease inhibitors‐treated adult microglia on the cognitive function of rats was evaluated by water maze. As shown in Figure 7a, b, the injection of protease inhibitors‐treated adult microglia significantly rescued increased escape latency and decreased platform crossing times of rats with TBI. So, declined cognitive function of rats after the induction of TBI was improved by protease inhibitors‐treated adult microglia.

## 4 DISCUSSION

The generation and development of TBI is a multifactorial and complex process. Primary injuries are brain tissue damages caused by external forces, including axonal shearing, contusions, and the formation of hemorrhage.^12^ As a consequence of these brain tissue damages, a series of biochemical and molecular changes are initiated and called secondary injuries, including cell apoptosis, inflammation response, ischemic necrosis, and excitotoxicity.^13^ The process of secondary injuries has critical role in injury expansion and brain tissue damage. Thus, a plenty of researches related to TBI focus on investigating the molecular mechanism of secondary injury cascade in order to alleviate tissue degeneration and cell death caused by TBI.

Among these secondary injuries caused by TBI, neuroinflammation is thought to be the major pathological process.^14^ Since the inflammatory response during TBI is generated without pathogenic stimulus, it is also called “sterile inflammation.”^15^ Neuroinflammation induced by TBI involves diverse cell types, such as astrocytes, microglia, peripheral immune cells, and cerebrovascular endothelial cells.^16^

As specialized immune cells in brain, microglia have phagocytic and antigen‐presenting abilities.^17^ The shape and structure of microglia are related to their activation state. In resting state, microglia have ramified morphology.^18^ In activation state, the morphology of microglia changes in to hypertrophic.^19^ During the pathogenesis of TBI, microglia are the first responders to trauma. Under normal homeostatic conditions, microglia exist in resting state and detect alterations in local environments through synthesizing/secreting signaling molecules.^20^ In response to the damage of brain tissue, microglia are activated and rapidly change between pro‐inflammatory phenotype (M1‐Like) and anti‐inflammatory phenotype (M2‐like).^21^, ^22^ M1‐like phenotype activated microglia mainly synthesize and secrete pro‐inflammatory cytokines and chemokines, such as IL‐1β, IL‐6, IL‐12, TNF‐α, and MCP‐1.^23^ These cytokines and chemokines regulate the plasticity of synapses, the release of neurotransmitter, and the excitability of neurons.^24^ Activated microglia with M2‐like phenotype promote anti‐inflammatory profiles and decrease neuroinflammation by enhanced phagocytic activity.^25^ Thus, the dynamic roles of microglia in neuroinflammation indicate that activated microglia control both the repair of tissues and the destruction of tissues. Csf1 and MCP‐1 are key factors for monocyte/macrophage recruitment to the injury site. In this research, we stained microglia and macrophage marker Iba1, without distinguishing microglia and recruited macrophages. Based on reduced levels of Csf‐1 and MCP‐1, we speculated that microglial transplantation might repress the recruitment of monocyte/macrophage to the lesion. FACS is an efficient tool to distinguish microglia (CD45low, CD11b+) and monocytes/macrophages (CD45high, CD11b+). However, we could not obtain enough neonatal animals in the limited time to collect enough samples for analyzing monocytes/macrophages, though it is an interesting question. It was a shortcoming of this research not to explore the function of microglial transplantation on monocyte/macrophage recruitment.

The activation of microglia in pediatric TBI models has not been fully investigated. In neonatal mouse TBI model, the degeneration of neurons and the activation of microglia have been proved to exist in the same area.^26^ In neonatal rats, microglial response induced by hypoxic–ischemic brain injury was stronger in 9‐day‐old animals compared with animals that were injured on postnatal day 30.^27^

Recently, a research has reported that the depletion of microglia disrupts scar‐free healing in neonatal mice with injured spinal cord and the transplantation of neonatal microglia enhanced axon regrowth and healing in spinal cord.^10^ Thus, we investigated whether the injection of neonatal microglia could also play a role in alleviating the damages caused by TBI in rat model.

One of the common features of TBI is abnormal cell apoptosis in central nervous system. In neuronal and glial cells, caspase‐3 upregulation triggers cell apoptosis and TBI pathology.^28^ Since activation of caspase‐3 is a well‐recognized hallmark of neuronal apoptosis, the protein level of cleaved caspase‐3 of injured cortex and ipsilateral hippocampus were analyzed in this research. Changed cleaved caspase‐3 level indicated that the injection of neonatal microglia significantly inhibited enhanced cell apoptosis in the brain of TBI rat model, while the injection of adult microglia had no effect. Astrocytes and microglia are considered key players in initiating an inflammatory response after injury.^9^ The astrocytosis and microgliosis in injured cortex and ipsilateral hippocampus of TBI rat model were significantly inhibited by the injection of neonatal microglia. Meanwhile, neonatal microglia also decreased the level of neuroinflammation‐related cytokines and chemokines in the brain of TBI model. The pathogenesis of TBI decreased the cognitive function of rats. The injection of neonatal microglia, but not adult microglia, improved the cognitive function of TBI rat model. Thus, in TBI rat model, the transplantation of neonatal microglia significantly alleviated the damages in brain tissue.

When compared with neonatal microglia, adult microglia had no influence on the pathogenesis of TBI in rats. In both mice and rats, neonatal animals are capable of scar‐free wound healing and spontaneous axon regrowth.^29^ It has been proved that microglia play dominant function in the reparative injury response in neonatal mice, exhibiting powerful inflammation‐resolution properties.^10^ In adult, the activation of microglia caused by injury is permanent. In contrast, initially activated neonatal microglia can go back to a homeostatic state rapidly and spontaneously. This temporal activation of neonatal microglia is crucial for the scar‐free wound‐healing process in spinal cord.^10^ The specific re‐establishing homeostasis function of neonatal microglia may be critical for alleviating the damages caused by TBI in brain tissue. A research in mice indicated that neonatal MG3 microglia promoted scar‐free wound healing through its specific molecular properties.^10^ First, neonatal microglia secrete fibronectin and generate the ligation of extracellular matrix. Second, compared with adult microglia, neonatal microglia express several extra peptidase inhibitors and inflammation‐relative molecules, including cystatin B (Cstb), stefins A1 (Stfa1), Serpinb6a, and annexin A1 (Anxa1). Thus, in rat, the different treatment effects of neonatal and adult microglia might also be caused by these different molecular properties.

In neonatal mouse model with spinal cord injury, neonatal microglia have a significant enrichment for anti‐inflammatory genes, including those associated with the activity of serine‐type and cysteine‐type endopeptidase inhibitors and phospholipase A2 inhibitors.^10^ Thus, adult microglia were treated by two chemical proteinase inhibitors: E64, a membrane‐permeable irreversible inhibitor of a wide range of cysteine peptidases, and serpinA3N, a serine protease inhibitor. Research has proved that adult microglia treated with a combination of E64 and serpinA3N also have specific molecular properties that promote scar‐free wound healing in spinal cord.^10^

In this research, we also investigated whether adult microglia could have the same effects as neonatal microglia in the brain of TBI rat model after being treated with E64 and serpinA3N. Results in this research demonstrated that protease inhibitors‐treated adult microglia could also inhibit cell apoptosis, reduce astrocytosis and microgliosis, and improve cognitive function in TBI rat model. Thus, our results suggest that re‐establishing homeostasis of adult microglia through proteinase inhibitors enhanced the effects of adult microglia on alleviating the damages caused by TBI in brain tissue.

Although we proved that both neonatal microglia and protease inhibitors‐treated adult microglia could inhibit the pathogenesis of TBI in rat model, the molecular mechanism during this process was still unknown. Classically, microglia primarily participate in immune responses and homeostasis regulation.^30^ Recently, research has demonstrated that microglia also have critical functions in central nervous system development, including inducing programmed neuronal death, promoting neuronal survival, and regulating synaptogenesis.^31^ In adult mice model with spinal cord injury, activated microglia accumulated in the region of lesion and were surrounded by activated astrocytes, fibroblasts, and basal lamina components. By contrast, in neonatal mice model, activated microglia number in the region of lesion was significantly reduced and the deposition of extracellular matrix components was also decreased.^10^ In the injured adult spinal cord, macrophages derived from blood monocytes accumulated and persistently existed in the lesion, while in neonatal spinal cord, accumulated monocyte‐derived macrophages were absent in several days.^10^ These indicated that the effects of neonatal microglia and protease inhibitors‐treated adult microglia in TBI rat model may also related to the functions in regulating extracellular matrix component deposition, macrophage accumulation, and central nervous system development. In this research, results indicated that the injection of neonatal microglia or protease inhibitors‐treated adult microglia was a potential therapeutic strategy for patients with TBI.

In this research, the effect of microglial translation was only evaluated at 5 days post injury. Thus, the temporal effect of microglial transplantation was not explored in this research. In a rat model of spinal cord injury, the hindlimb motor function was enhanced by microglial cell transplantation at 2, 3, 4, 6, and 8 weeks after surgery. Meanwhile, the expression of CD68 and OX42 in transplanted microglial cells maintained at a relatively higher level at 2 days, 1 week and 2 weeks, and then decreased at 3 weeks and 4 weeks after transplantation. It is generally accepted that acute microglial response has neuroprotective effects, while the chronically activated state of microglial enhances the negative effects of repetitive injuries on neurological health.^32^ Thus, microglia cells activate on the early phase to exhibit positive effect on injury recovery and change into resting state to avoid the toxic effect of excessive activation.^33^ In the brain of TBI rat model, the transplantation of microglial might also have an early active state and a late resting state. The details in this process should be investigated in future work.

In conclusion, the transplantation of either neonatal microglia or adult microglia treated with peptidase inhibitors significantly inhibited the pathogenesis of TBI in rat model, while untreated adult microglia showed no significant effect.

## CONFLICT OF INTERESTS

The authors declare that they have no competing interests.

## AUTHOR CONTRIBUTIONS

**Ping Zheng:** Data curation (lead); formal analysis (equal); validation (lead); writing – original draft (equal). **Qingke Bai:** Conceptualization (equal); data curation (equal); investigation (equal); validation (equal); writing – original draft (equal). **Jiugeng Feng:** Data curation (equal); investigation (equal); methodology (equal); validation (equal); writing – original draft (equal). **Bing Zhao:** Conceptualization (equal); data curation (equal); validation (equal); writing – original draft (equal). **Jian Duan:** Data curation (equal); investigation (equal); validation (equal); writing – original draft (equal). **Lin Zhao:** Data curation (equal); investigation (equal); validation (equal); writing – original draft (equal). **Ning Liu:** Data curation (equal); formal analysis (equal); investigation (equal); validation (equal); writing – original draft (equal). **Dabin Ren:** Data curation (equal); funding acquisition (equal); investigation (equal); validation (equal); writing – original draft (equal); writing – review and editing (equal). **Shufeng Zou:** Data curation (equal); funding acquisition (equal); investigation (equal); validation (equal); writing – original draft (equal); writing – review and editing (equal). **Wei Chen:** Conceptualization (lead); data curation (lead); formal analysis (lead); investigation (lead); validation (lead); visualization (lead); writing – original draft (lead); writing – review and editing (lead).

## Data Availability

The data will be made available upon reasonable request.

## References

[1] Najem D , Rennie K , Ribecco‐Lutkiewicz M , et al. Traumatic brain injury: classification, models, and markers. Biochem Cell Biol. 2018;96(4):391‐406. 29370536 10.1139/bcb-2016-0160

[2] Kenne E , Erlandsson A , Lindbom L , Hillered L , Clausen F . Neutrophil depletion reduces edema formation and tissue loss following traumatic brain injury in mice. J Neuroinflammation. 2012;9:17. 22269349 10.1186/1742-2094-9-17 PMC3292978

[3] Ladak AA , Enam SA , Ibrahim MT . A review of the molecular mechanisms of traumatic brain injury. World Neurosurg. 2019;131:126‐132. 31301445 10.1016/j.wneu.2019.07.039

[4] McGinn MJ , Povlishock JT . Pathophysiology of traumatic brain injury. Neurosurg Clin N Am. 2016;27(4):397‐407. 27637392 10.1016/j.nec.2016.06.002

[5] Webster KM , Sun M , Crack P , O'Brien TJ , Shultz SR , Semple BD . Inflammation in epileptogenesis after traumatic brain injury. J Neuroinflammation. 2017;14(1):10. 28086980 10.1186/s12974-016-0786-1 PMC5237206

[6] Corrigan F , Mander KA , Leonard AV , Vink R . Neurogenic inflammation after traumatic brain injury and its potentiation of classical inflammation. J Neuroinflammation. 2016;13(1):264. 27724914 10.1186/s12974-016-0738-9 PMC5057243

[7] Younger D , Murugan M , Rama Rao KV , Wu LJ , Chandra N . Microglia receptors in animal models of traumatic brain injury. Mol Neurobiol. 2019;56(7):5202‐5228. 30554385 10.1007/s12035-018-1428-7

[8] Hernandez‐Ontiveros DG , Tajiri N , Acosta S , Giunta B , Tan J , Borlongan CV . Microglia activation as a biomarker for traumatic brain injury. Front Neurol. 2013;4:30. 23531681 10.3389/fneur.2013.00030 PMC3607801

[9] Karve IP , Taylor JM , Crack PJ . The contribution of astrocytes and microglia to traumatic brain injury. Br J Pharmacol. 2016;173(4):692‐702. 25752446 10.1111/bph.13125 PMC4742296

[10] Li Y , He X , Kawaguchi R , et al. Microglia‐organized scar‐free spinal cord repair in neonatal mice. Nature. 2020;587(7835):613‐618. 33029008 10.1038/s41586-020-2795-6 PMC7704837

[11] Chen W , Zhao L , Zhang J , et al. Elevated expression of miR‐302 cluster improves traumatic brain injury by inhibiting phosphorylation of connexin43 via ERK signaling. J Chem Neuroanat. 2019;99:1‐8. 31096001 10.1016/j.jchemneu.2019.05.003

[12] Werner C , Engelhard K . Pathophysiology of traumatic brain injury. Br J Anaesth. 2007;99(1):4‐9. 17573392 10.1093/bja/aem131

[13] Greve MW , Zink BJ . Pathophysiology of traumatic brain injury. Mt Sinai J Med. 2009;76(2):97‐104. 19306379 10.1002/msj.20104

[14] Cederberg D , Siesjo P . What has inflammation to do with traumatic brain injury? Childs Nerv Syst. 2010;26(2):221‐226. 19940996 10.1007/s00381-009-1029-x

[15] Rock KL , Latz E , Ontiveros F , Kono H . The sterile inflammatory response. Annu Rev Immunol. 2010;28:321‐342. 20307211 10.1146/annurev-immunol-030409-101311 PMC4315152

[16] DiSabato DJ , Quan N , Godbout JP . Neuroinflammation: the devil is in the details. J Neurochem. 2016;139 (Suppl 2):136‐153. 26990767 10.1111/jnc.13607 PMC5025335

[17] Hickey WF , Kimura H . Perivascular microglial cells of the CNS are bone marrow‐derived and present antigen in vivo. Science. 1988;239(4837):290‐292. 3276004 10.1126/science.3276004

[18] Glenn JA , Ward SA , Stone CR , Booth PL , Thomas WE . Characterisation of ramified microglial cells: detailed morphology, morphological plasticity and proliferative capability. J Anat. 1992;180 (Pt 1):109‐118. 1452465 PMC1259614

[19] Tambuyzer BR , Ponsaerts P , Nouwen EJ . Microglia: gatekeepers of central nervous system immunology. J Leukoc Biol. 2009;85(3):352‐370. 19028958 10.1189/jlb.0608385

[20] Hughes AN , Appel B . Microglia phagocytose myelin sheaths to modify developmental myelination. Nat Neurosci. 2020;23(9):1055‐1066. 32632287 10.1038/s41593-020-0654-2 PMC7483351

[21] Habib P , Slowik A , Zendedel A , Johann S , Dang J , Beyer C . Regulation of hypoxia‐induced inflammatory responses and M1‐M2 phenotype switch of primary rat microglia by sex steroids. J Mol Neurosci. 2014;52(2):277‐285. 24163150 10.1007/s12031-013-0137-y

[22] Yao X , Liu S , Ding W , et al. TLR4 signal ablation attenuated neurological deficits by regulating microglial M1/M2 phenotype after traumatic brain injury in mice. J Neuroimmunol. 2017;310:38‐45. 28778443 10.1016/j.jneuroim.2017.06.006

[23] Norden DM , Trojanowski PJ , Villanueva E , Navarro E , Godbout JP . Sequential activation of microglia and astrocyte cytokine expression precedes increased Iba‐1 or GFAP immunoreactivity following systemic immune challenge. Glia. 2016;64(2):300‐316. 26470014 10.1002/glia.22930 PMC4707977

[24] Schumann J , Alexandrovich GA , Biegon A , Yaka R . Inhibition of NR2B phosphorylation restores alterations in NMDA receptor expression and improves functional recovery following traumatic brain injury in mice. J Neurotrauma. 2008;25(8):945‐957. 18721106 10.1089/neu.2008.0521 PMC2946870

[25] Laffer B , Bauer D , Wasmuth S , et al. Loss of IL‐10 promotes differentiation of microglia to a M1 phenotype. Front Cell Neurosci. 2019;13:430. 31649508 10.3389/fncel.2019.00430 PMC6794388

[26] Tong W , Igarashi T , Ferriero DM , Noble LJ . Traumatic brain injury in the immature mouse brain: characterization of regional vulnerability. Exp Neurol. 2002;176(1):105‐116. 12093087 10.1006/exnr.2002.7941

[27] Ferrazzano P , Chanana V , Uluc K , et al. Age‐dependent microglial activation in immature brains after hypoxia‐ischemia. CNS Neurol Disord Drug Targets. 2013;12(3):338‐349. 23469850 10.2174/1871527311312030007 PMC3674227

[28] Mattson MP . Apoptosis in neurodegenerative disorders. Nat Rev Mol Cell Biol. 2000;1(2):120‐129. 11253364 10.1038/35040009

[29] Miya D , Giszter S , Mori F , Adipudi V , Tessler A , Murray M . Fetal transplants alter the development of function after spinal cord transection in newborn rats. J Neurosci. 1997;17(12):4856‐4872. 9169544 10.1523/JNEUROSCI.17-12-04856.1997 PMC6573335

[30] Nayak D , Roth TL , McGavern DB . Microglia development and function. Annu Rev Immunol. 2014;32:367‐402. 24471431 10.1146/annurev-immunol-032713-120240 PMC5001846

[31] Ueno M , Fujita Y , Tanaka T , et al. Layer V cortical neurons require microglial support for survival during postnatal development. Nat Neurosci. 2013;16(5):543‐551. 23525041 10.1038/nn.3358

[32] Simon DW , McGeachy MJ , Bayir H , Clark RS , Loane DJ , Kochanek PM . The far‐reaching scope of neuroinflammation after traumatic brain injury. Nat Rev Neurol. 2017;13(3):171‐191. 28186177 10.1038/nrneurol.2017.13 PMC5675525

[33] Kou D , Li T , Liu H , et al. Transplantation of rat‐derived microglial cells promotes functional recovery in a rat model of spinal cord injury. Braz J Med Biol Res. 2018;51(10):e7076. 30066721 10.1590/1414-431X20187076 PMC6075796

